# Mining of favorable alleles for seed reserve utilization efficiency in *Oryza sativa* by means of association mapping

**DOI:** 10.1186/s12863-020-0811-3

**Published:** 2020-01-16

**Authors:** Nour Ali, Dalu Li, Moaz S. Eltahawy, Dina Abdulmajid, Lal Bux, Erbao Liu, Xiaojing Dang, Delin Hong

**Affiliations:** 1 Nanjing Agricultural University, Nanjing, 210095 China; 2 State Key Laboratory of Crop Genetics and Germplasm Enhancement, Nanjing Agricultural University, Nanjing, 210095 China; 3 Laboratory of Crop Production and Multiplication, Field Crops Research Department, Agricultural Faculty, Damascus University, Damascus, Syria; 4 Laboratory of Crop Genetics and Germplasm Enhancement, Field Crops Research Department, Agricultural Faculty, Damascus University, Damascus, Syria; 5 Agronomy Department, Faculty of Agriculture, Zagazig University, Zagazig, Sharqia 44519 Egypt; 6 Rice Research and Training Center, Field Crops Research Institute, Agricultural Research Center, Kafr El-Sheikh, 33717 Egypt

**Keywords:** *Oryza sativa*, Seed reserve utilization efficiency, Association mapping, Favorable alleles, Direct seeding

## Abstract

**Background:**

Wet direct-seeded rice is a possible alternative to conventional puddled transplanted rice; the former uses less water and reduces labor requirements. Improving seed reserve utilization efficiency (SRUE) is a key factor in facilitating the application of this technology. However, the QTLs controlling this trait are poorly investigated. In this study, a genome-wide association study (GWAS) was conducted using a natural population composed of 542 accessions of rice (*Oryza sativa* L.) which were genotyped using 266 SSR markers. Large phenotypic variations in SRUE were found in the studied population.

**Results:**

The average SRUE over 542 accessions across two years (2016 and 2017) was 0.52 mg.mg^− 1^, ranging from 0.22 mg.mg-^1^ to 0.93 mg.mg^− 1^, with a coefficient of variation of 22.66%. Overall, 2879 marker alleles were detected in the population by 266 pairs of SSR markers, indicating a large genetic variation existing in the population. Using general linear model method, 13 SSR marker loci associated with SRUE were detected and two (RM7309 and RM434) of the 13 loci, were also detected using mixed linear model analyses, with percentage of phenotypic variation explained (PVE) greater than 5% across two years. The 13 association loci (*P* < 0.01) were located on all chromosomes except chromosome 11, with PVE ranging from 5.05% (RM5158 on chromosome 5) to 12% (RM297 on chromosome 1). Association loci RM7309 on chromosome 6 and RM434 on chromosome 9 revealed by both models were detected in both years. Twenty-three favorable alleles were identified with phenotypic effect values (PEV) ranging from 0.10 mg.mg^− 1^ (RM7309–135 bp on chromosome 9) to 0.45 mg.mg^− 1^ (RM297–180 bp on chromosome 2). RM297–180 bp showed the largest phenotypic effect value (0.44 mg.mg^− 1^ in 2016 and 0.45 mg.mg^− 1^ in 2017) with 6.72% of the accessions carrying this allele and the typical carrier accession was Manyedao, followed by RM297–175 bp (0.43 mg.mg^− 1^ in 2016 and 0.44 mg.mg^− 1^ in 2017).

**Conclusion:**

Nine novel association loci for SRUE were identified, compared with previous studies. The optimal parental combinations for pyramiding more favorable alleles for SRUE were selected and could be used for breeding rice accessions suitable for wet direct seeding in the future.

## Background

Rice (*Oryza sativa* L.) is the basic daily food for billions of people worldwide. It is considered to be the oldest domesticated grain (~ 10,000 years) and grown in the largest single use of land, covering 9% of the earth’s arable land (158.8 million hectares). Asia holds over 90% of the world’s production of rice, with China (208.6 million metric tons), India (109.15 million metric tons) and Indonesia (74.2 million metric tons) producing the bulk of the continental production [1].

To keep up with the accelerated development of the economy, labor force migration, the decline in fresh water quality and volume, and changing crop cultivation practices and mechanization, adopting direct seeding technology in rice crop cultivation has become a necessary transformation. Wet direct seeding involves the sowing of pre-germinated seeds with a radical variation in size, from 1 to 3 mm on or into puddle soil and is proving to be a promising technology. The essence of this technology is the seedling vigor which can be considered as the product of three components: (1) initial seed weight, (2) the fraction of seed reserves which are mobilized, and (3) the conversion efficiency of mobilized seed reserves to seedling tissues [2, 3]. Seed reserve utilization efficiency (SRUE) is an important characteristic of seedling vigor, since seedling growth can be limited by decreased mobilization of seed reserve and/or the conversion efficiency of mobilized seed reserves.

The physiological characteristics of SRUE had been evaluated in different crops such as Lithocarpus densiflora [4], wheat [2, 5], maize [6, 7], soybean [3] and sorghum [8]. As for rice, Cheng et al. (2013) identified thirteen additive QTLs (on chromosomes 2, 4, 8 and 12) and two pairs of epistatic QTLs (on chromosomes 7, 8 and 12) for SRUE using the recombinant inbred lines (RILs) derived from Jiucaiqing and IR26 and found qSRUE4.3 explained more than 20% of the total phenotypic variance [9]. Cheng et al. (2015) found that α-amylase (*OsAmy3B*, *OsAmy3C*, and *OsAmy3E*) and sucrose synthase (*OsSus2*, *OsSus3*, and *OsSus4*) genes might be involved in seed reserve utilization [10]. However, linkage mapping is limited by the fact that only two alleles can be studied at any given locus in bi-parental crosses of inbred lines.

Association mapping based on linkage disequilibrium (LD) using natural populations for QTL analysis is widely used in plant kingdom, as a popular method to search for, and discover favorable alleles for many traits, including agronomic traits [11–22] and seed vigor traits [23–25]. However, no studies have been undertaken to discover favorable alleles for SRUE in natural rice populations. The aims of this study were (1) to investigate the phenotypic variation of SRUE trait in the natural population composed of 542 accessions in *Oryza sativa*. (2) to mine favorable alleles of SRUE for improving accessions suitable for wet direct sowing cultivation by machine, and (3) to provide optimal parental combinations for pyramiding excellent alleles into a single plant.

## Results

### Phenotypic variations of SRUE in the natural population

The mean value, standard deviation, skewness, and kurtosis for SRUE measured in 542 rice accessions in 2016 were shown in Table 1. Variance analysis showed that there were significant genetic differences among 542 rice accessions at the probability level of α = 0.01. The average of SRUE over 542 accessions was 0.52 mg.mg^− 1^ ranging from 0.21 mg.mg^− 1^ to 0.96 mg.mg^− 1^, with a coefficient of variation of 23.80%. 31.55% of total accessions had SRUE values larger than 0.55 mg.mg^− 1^ and 30.44% of total accessions had SRUE values greater than 0.65 mg.mg^− 1^. The generalized heritability of SRUE was 99.72%, indicating that the variation of SRUE trait was less affected by the environment. The mean, range of phenotypic values, generalized heritability and coefficient of variation of SRUE in 2017 were similar to those of 2016 (Table 1). These results indicated that there exists abundant genetic variation of SRUE in this natural population used.

**Table 1:** Descriptive statistics of SRUE^*^ (mg.mg^− 1^) in 542 rice accessions across 2 years

Items	2016	2017
Mean (mg.mg^− 1^)	0.518	0.526
Minimum (mg.mg^− 1^)	0.209	0.242
Maximum (mg.mg^− 1^)	0.962	0.917
Standard Deviation (mg.mg^− 1^)	0.129	0.113
Skewness	0.595	0.536
Kurtosis	0.253	0.165
CV (%)	23.80	21.52
*H*^*2*^_*B*_ (%)	99.72	99.89

*SRUE: seed reserve utilization efficiency

### Molecular marker allele diversity of SSR loci in the natural population

The genetic diversity of all 542 rice accessions was evaluated using 266 SSR markers distributed in the whole genome. Different sizes of DNA fragments (Additional file 1: Figure S1) amplified by the same pair of SSR primers among the 542 accessions were regarded as allelic variation fragments of the pair of primers. 2879 alleles were detected in 542 rice accessions. The average number of alleles per SSR locus was 10.82. The variation ranges were from 2 (RM437 on chromosome5, RM7163 on chromosome11) to 38 (RM3428 on chromosome11) (Additional file 5: Table S1). The average genetic diversity per locus over all the 266 SSR loci was 0.74 and the variation range was 0.08 (RM7163 on chromosome11) - 0.9506 (RM3428 on chromosome11), and was mainly distributed between 0.75 and 0.95. The average PIC value was 0.71, ranging from 0.08 (RM7163 on chromosome11) to 0.95 (RM3428 on chromosome11). PIC represents an indicator of the degree of microsatellite DNA variation, reflecting the level of microsatellite DNA polymorphism. Two hundred and thirty-one SSR loci (occupied 86.84% of all SSR loci used) showed highly informative (PIC > 0.5), 29 loci (10.90%) moderately informative (0.5 > PIC > 0.25), and 6 loci (2.25%) slightly informative (PIC < 0.25) (Additional file 5: Table S1).

### Genetic structure of the population used

**Fig. 1:**
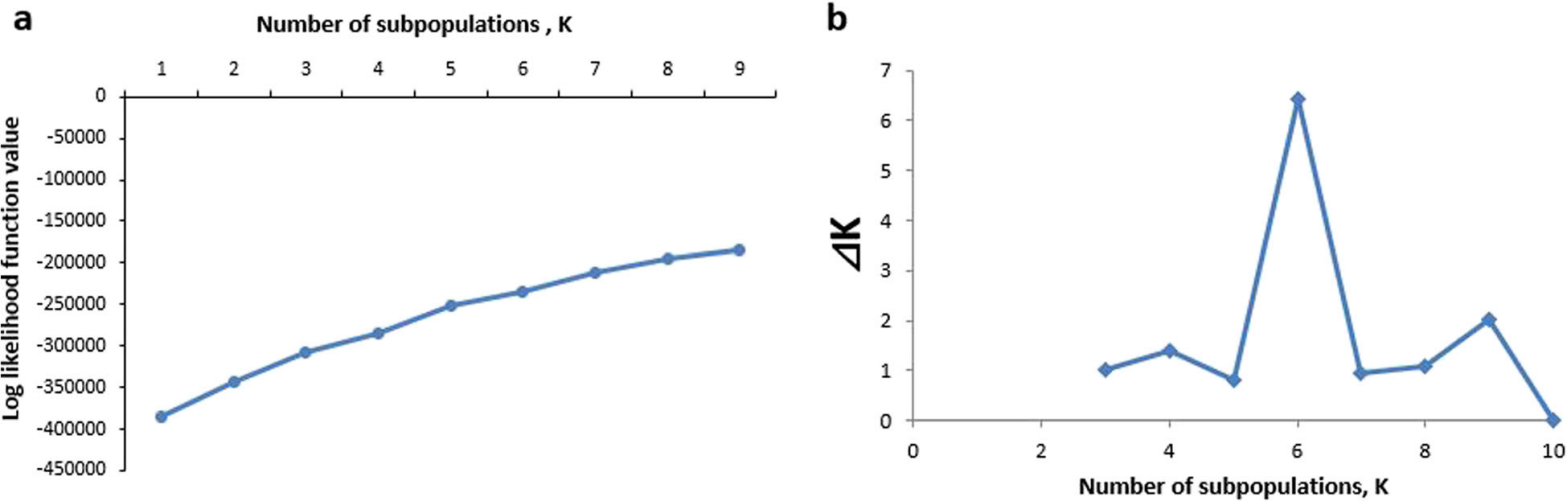
Changes in the number of subpopulations with **a** the log-likelihood function value, **b** with ∆K values

**Fig. 2:**
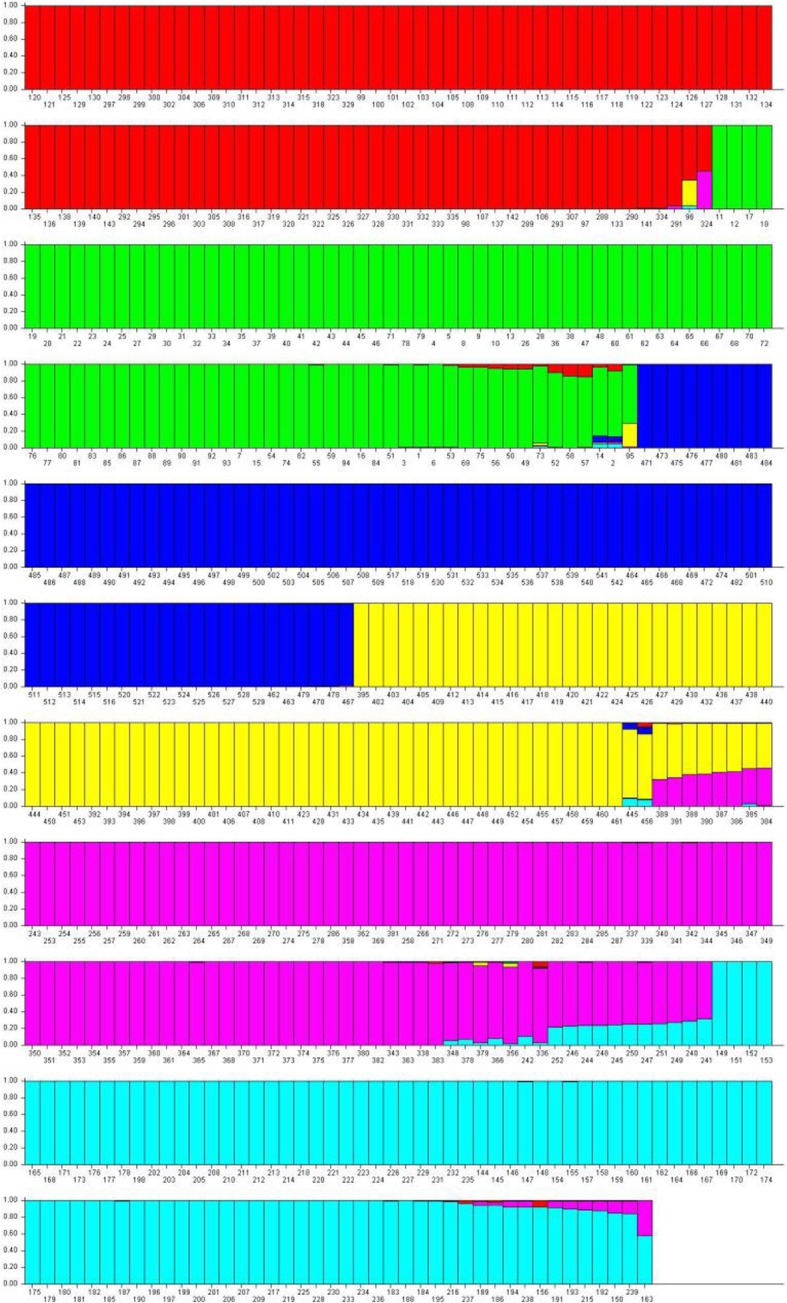
All 542-rice variety belonging to six subpopulations defined by STRUCRURE software. Identified sub-populations are POP1 (red color), POP2 (green color), POP3 (navy blue color), POP4 (yellow color), POP5 (purple color), POP6 (light blue color)

Using SSR marker molecular data and STRUCTURE 2.2 software to analyze the genetic structure of the total population of rice accessions, it was found that the log-likelihood function values increase with the number of sub-populations (Fig. 1a). The number of subpopulation k value is then determined by ∆K value (the rate of change of the log-likelihood values on successive K values) calculated using the analytical method of Evanno et al. (2005) [26]. Fig. 1b shows that ∆K value reached maximum at K = 6. Therefore, the entire population can be divided into 6 sub-populations. Each accession was sorted into the corresponding subpopulation according to the obtained Q value (Q > 0.9) (Additional file 6: Table S2). Based on the Q value the 542 rice accessions were grouped into six subpopulations, that is, POP1 (94 accessions), POP2 (89 accessions), POP3 (81 accessions), POP4 (68 accessions), POP5 (83 accessions), POP6 (91 accessions) and an admix group (36 accessions). The posterior probability value of each accession belonging to the six subpopulations is shown in Fig. 2.

Furthermore, it was found that each subpopulation is consist of accessions with the same geographic origin. For example, POP1 accessions were from Jiangsu province, China and Vietnam (*Tej* and *Indica*), POP2 has accessions most of which are modern breeds in north-central Jiangsu (*Tej*), POP3 contains accessions with the majority of quality accessions in Jiangsu Province (*Tej*), POP4 contains accessions which were tall, late-maturing accessions and a small number of northeast accessions in the Taihu Lake Basin (*Tej*), POP5 accessions were mainly from Vietnam (*Indica*) and POP6 contains accessions of Taihu tall, early maturing accessions (*Tej*).

**Fig. 3:**
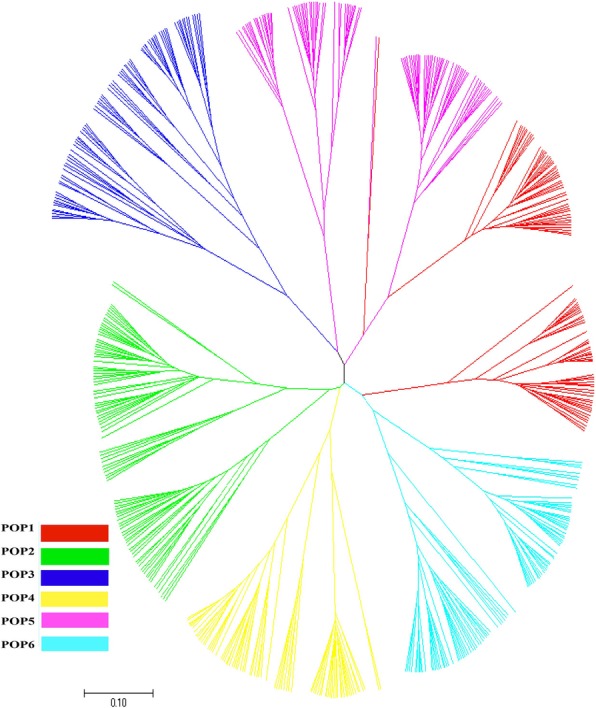
Neighbor-joining tree for the 542 accessions generated using Nei’s genetic distance

In order to verify the reliability of population genetic structure partitioning, a neighbor-joining (NJ) clustering map was constructed, for the total population of 542 rice accessions by using Nei’s (1983) genetic distance [27], calculated by software POWERMARKER 3.25 and observed by software MEGA 4.0. The NJ cluster map (Fig. 3) shows that the total population of the 542 rice accessions is clearly clustered into 6 subpopulations. This is consistent with the structural analysis based on the STRUCTURE model, indicating that the total population of this study was divided into 6 subpopulations with good reliability.

### Genetic differentiation among subpopulations

The average genetic differentiation index *F*_st_ among the six subpopulations was 0.36, with the *F*_st_ for each locus ranging 0.008 for RM5479 on chromosome 12 to 0.88 for RM218 on chromosome 3. Pairwise comparisons based on *F*_st_ values can reflect the standard genetic distance between two populations [28]. *F*_st_ values ranged from 0.26 (POP1 and POP5) to 0.42 (POP3 and POP4), and the corresponding standard genetic distance between the two subpopulations ranged from 0.45 (POP1 and POP5) to 0.69 (POP3 and POP4) (Table 2). AMOVA indicated that 64.42% of the total genetic variation occurred among the subpopulations, whereas 35.58% occurred among the individuals within the subpopulations (Additional file 7: Table S3). These results indicate the existence of a high degree of genetic differentiation across the six subpopulations.

**Table 2:** Pairwise estimates of *F*_st_ and Nei’s genetic distance among the 6 subpopulations

Subpopulations	POP1	POP2	POP3	POP4	POP5	POP6
POP1	–	0.50	0.65	0.50	**0.45**	0.46
POP2	0.31	–	0.59	0.47	0.58	0.51
POP3	0.37	0.37	–	**0.69**	0.62	0.65
POP4	0.35	0.35	**0.42**	–	0.55	0.52
POP5	**0.26**	0.35	0.36	0.38	–	0.49
POP6	0.30	0.36	0.41	0.40	0.36	–

Nei’s genetic distance estimates appear above the diagonal, and pairwise *F*_*st*_ appear below the diagonal. All *F*_*st*_ values are significant (*P* < 0.01)

### Linkage disequilibrium analysis

Among the 35,245 pairs of loci generated by 266 SSR loci, 23,081 pairs showed significant LD (based on *D*^′^ value, *P* < 0.01), of which 1919 pairs (5.44%) were intra-chromosomal pairs of SSR loci. Table 3 shows the percentage of significant LD locus pairs to the total number of pairwise loci in each subpopulation, of which POP1 is the highest (4.78%), while the POP6 is the lowest (3.13%). From the average of *D*^′^ values, POP1 was the highest (0.83), followed by POP5 (0.81) while POP3 was the lowest (0.58). Further regression analysis of *D*^′^ values and genetic distances of syntenic (intra-chromosome) marker pairs revealed that the attenuation of *D*^′^ values in each subpopulation was in accordance with the equation *y* = *blnx* + *c* (Additional file 2: Figure. S2). Therefore, the minimum distances of LD decay (*D*^′^ < 0.5) of each subpopulation were determined to be 58.08 cM (POP1), 27.75 cM (POP2), 17.57 cM (POP3), 19.23 cM (POP4), 34.05 cM (POP5) and 30.36 cM (POP6). It is clear that POP3 exhibited the highest decay velocity with the shortest decay distance, while POP1 showed the lowest decay velocity among the six sub-populations.

**Table 3:** D’ of LD for pairwise SSR loci each subpopulation

Subpopulation	Number of significant LD locus pairs ^a^	Number of significant LD locus pairs/Total number of locus pair (%) ^b^	Frequency of D’ value (*P* < 0.01)					Mean of D’ ^c^
			0–0.2	0.2–0.4	0.41–0.6	0.61–0.8	0.81–1.0	
POP1	1683	4.78	140	394	455	436	258	0.834
POP2	1350	3.83	115	231	306	315	397	0.705
POP3	1468	4.17	220	173	486	324	265	0.580
POP4	1599	4.54	128	329	507	212	423	0.679
POP5	1508	4.28	100	231	536	371	270	0.811
POP6	1103	3.13	112	182	293	236	190	0.741

*a LD means linkage disequilibrium

b Ratio between the number of significant LD locus pairs and total number of locus pairs

c D′ means standardized disequilibrium coefficients

### Detection of association loci

In total, thirteen SSR marker loci (with PVE > 5%) associated with SRUE were detected in both 2016 and 2017 by GLM and two of them were also detected by MLM in both years. The 13 marker loci were distributed on all chromosomes except chromosome 11. The percentage of phenotypic variation explained by single individual locus ranged from 5.03 to 12.01% in 2016 and 5.07 to 11.98% in 2017 respectively (Table 4). RM 297 on chromosome 1 explained the maximum phenotypic variation, viz. 12.01% in 2016 and 11.98% in 2017, respectively, followed by RM184 on chromosome 10 located at 41.6 cM (7.2% in 2016 and 7.32% in 2017) and the lowest was RM5158 on chromosome 5 located at 144.9 cM (5.03 and 5.07% in 2016 and 2017 respectively) (Table 4).

**Table 4:** SSR marker loci associated with SRUE (PVE > 5%) and percentage of phenotypic variation explained by the locus derived from 266 markers and 506 rice accessions

No#	Marker	Chrom	Position (cM)	2016			2017		
				*P* Value	PVE^a^ (%)	FDR^b^	*P* Value	PVE^a^ (%)	FDR^b^
1	RM128	1	123.2	0.003	5.85	0.003	0.003	5.91	0.003
2	RM297	1	126.5	0.009	12.01	0.009	0.009	11.98	0.009
3	RM525	2	143.7	0.007	6.40	0.007	0.007	6.42	0.007
4	RM232	3	76.7	0.003	5.61	0.003	0.003	5.63	0.003
5	RM3471	4	16.7	0.007	6.94	0.007	0.007	6.90	0.007
6	RM5818	5	144.9	0.002	5.03	0.002	0.002	5.07	0.002
7	**RM7309**	6	100.3	0.002	6.25	0.001	0.002	6.24	0.001
				0.001	7.18	7.23E-04	0.001	7.10	9.06E-04
8	RM3138	6	110.6	0.001	5.20	0.001	0.001	5.21	0.001
9	RM3589	7	89.8	0.002	5.49	0.002	0.002	5.51	0.002
10	RM281	8	128.1	0.006	5.62	0.006	0.007	5.55	0.007
11	**RM434**	9	57.7	0.002	5.24	0.001	0.002	5.22	0.002
				0.003	5.51	2.82E-03	0.003	5.52	2.73E-03
12	RM184	10	41.6	0.009	7.20	0.009	0.008	7.32	0.008
13	RM5746	12	39.4	0.007	5.94	0.007	0.008	5.85	0.008

*a PVE: Percentage of phenotypic variation explained

b FDR, False discovery rate

Markers in bold font means the common loci identified by both GLM and MLM in this study and the rest by GLM only

Among the 13 SSR association loci detected by GLM method, RM7309 on chromosome 6 and RM434 on chromosome 9, were also detected by MLM method associated with SRUE (Table 4). RM7309 had the higher contribution rate (viz 7.18% in 2016 and 7.10% in 2017, respectively) than those of RM434 (5.51% in 2016 and 5.52% in 2017, respectively). Compared with previous studies, 9 out of 13 loci (including RM434 detected by both GLM and MLM) are novel for SRUE (http://www.gramene.org/) (Additional file 8: Table S4).

### Discovery of favourable alleles

In this study, the alleles with positive effects are considered favorable alleles for SRUE. Table 5 shows a summary of favorable alleles of the significant association loci and their typical carriers for SRUE. In total, 23 favorable alleles with phenotypic effect value (PEV) more than 0.1 mg.mg^− 1^ for SRUE were detected across 506 rice accessions (Table 5). RM297–180 bp allele on chromosome 1showed the largest phenotypic effect (0.44 mg.mg^− 1^ in 2016 and 0.45 mg.mg^− 1^ in 2017), and 34 accessions (6.72%) carried this excellent allele, with Manyedao as the typical carrier. Fifty- eight accessions (11.46%) carried the excellent alleleRM297–175 bp, with Daniaodao as a typical carrier (Additional file 6: Table S2, Table 5). Excellent allele RM184–225 bp was carried by 30 (5.93%) accessions, with Yandao6 as a typical carrier. Excellent allele RM184–215 bp was carried by 51 (10.08%) accessions, with Daniaoda as a typical carrier. 30 accessions (5.93%) possessed an excellent alleleRM184–205 bp, with Manyedao as a typical carrier. 19 accessions (3.75%) possessed an excellent alleleRM7309–135 bp, which showed the smallest phenotypic effect (0.11 mg.mg^− 1^ in 2016 and 0. 10 mg.mg^− 1^ in 2017), with Manyedao as a typical carrier.

**Table 5:** Favorable alleles, their effects and typical carriers for SRUE of the 13 loci detected across 506 rice accessions in 2016 and 2017 (listed in descending order of phenotypic effect values)

Marker-allele(bp)	Phenotypic effect value (mg.mg^−1^)			Typical carrier
	2016	2017	Mean	
RM297–180	0.449	0.450	0.450	Manyedao
RM297–175	0.437	0.444	0.441	Daniaodao
RM184–225	0.432	0.430	0.431	Yandao6
RM184–215	0.413	0.418	0.416	Daniaodao
RM184–205	0.376	0.383	0.379	Manyedao
RM297–205	0.319	0.337	0.328	Youzhiyueguang
RM184–270	0.299	0.316	0.307	Youzhiyueguang
RM281–140	0.153	0.147	0.150	Yandao6
RM434–150	0.148	0.142	0.145	Yue40
RM5746–195	0.139	0.132	0.136	Yandao6
RM3589–210	0.141	0.131	0.136	Yue77
RM3471–110	0.1367	0.1297	0.1332	Yue40
RM5818–145	0.1365	0.1245	0.1305	Yue40
RM525–100	0.1338	0.1239	0.1289	Yue7
RM434–155	0.1311	0.126	0.1284	Yue77
RM5746–170	0.130	0.124	0.1270	Manyedao
RM3589–85	0.124	0.115	0.119	Yandao6
RM525–110	0.122	0.113	0.118	Jiaobaiyeqing
RM309–160	0.119	0.111	0.115	Yue77
RM3589–220	0.117	0.111	0.114	Yue40
RM3471–170	0.1154	0.110	0.113	Eyingbaijingdao
RM232–115	0.116	0.108	0.112	Yue77
RM7309–135	0.106	0.096	0.101	Manyedao

Locus-allele in bold font are identified by both GLM and MLM in this study and the rest by GLM only

### Excellent combination designs for improving SRUE

**Fig. 4:**
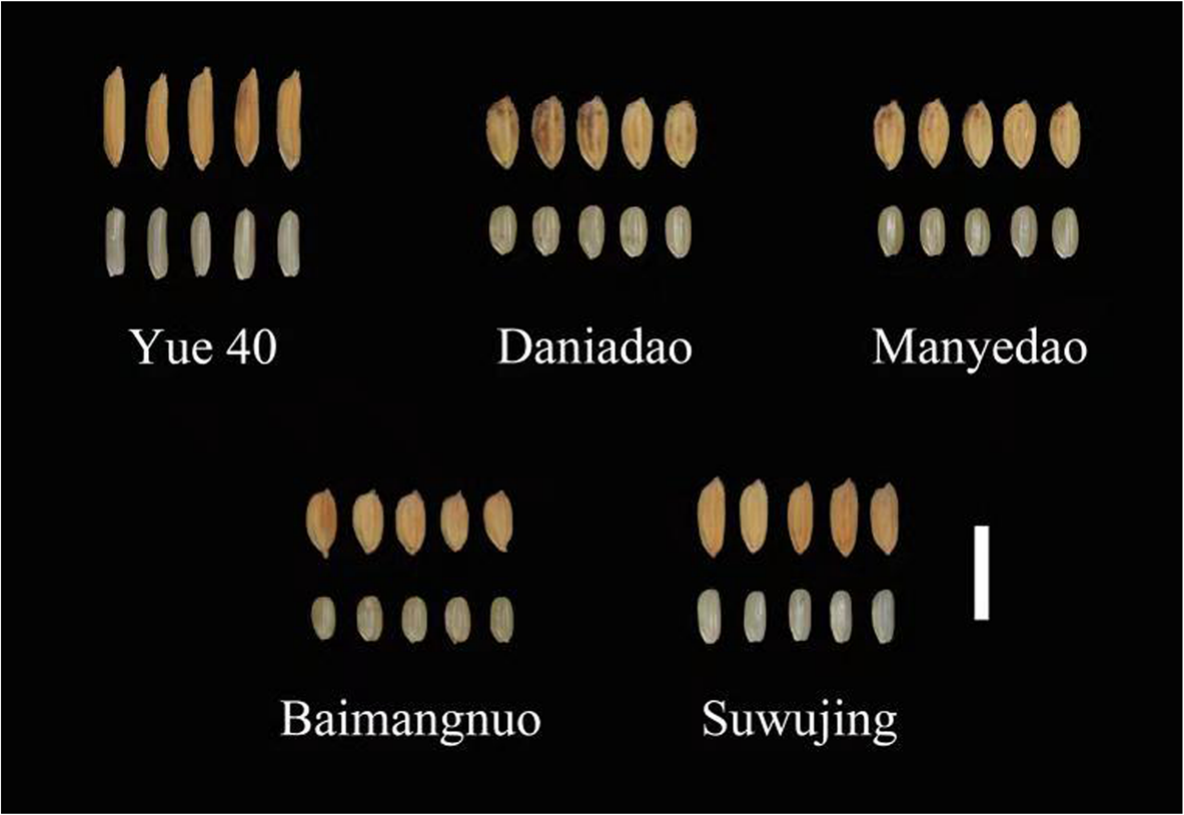
Un-hulled grains (above) and brown rice (down) of the favorable parents for improving of SRUE trait. Bar, 10 mm

**Fig. 5:**
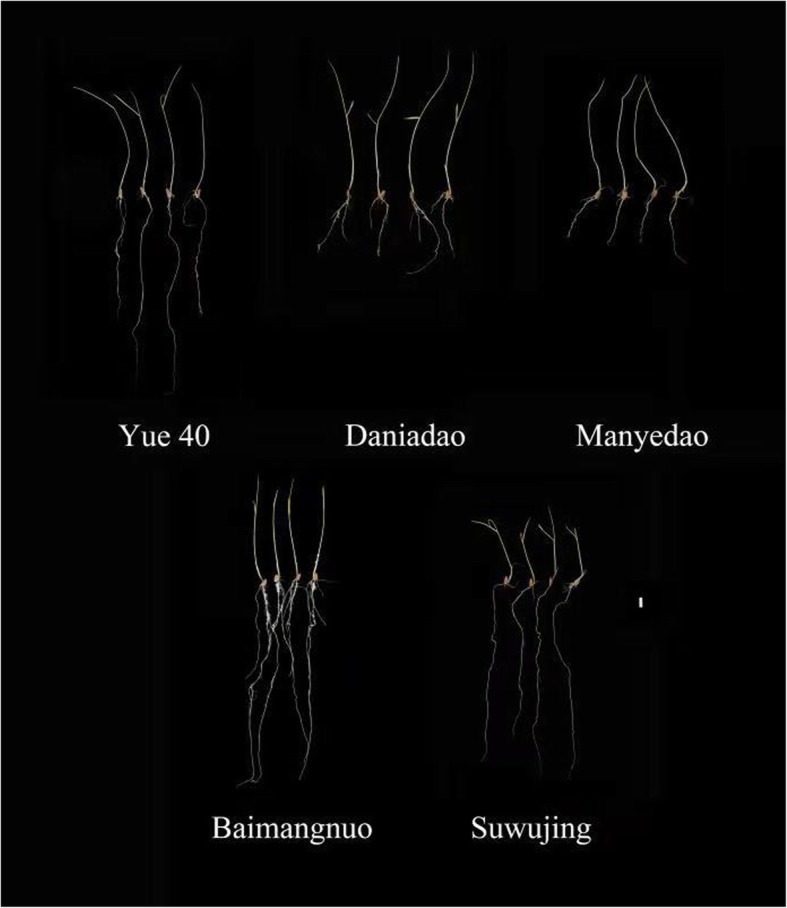
Etiolated seedlings of 10-days old of the favorable parents for improving of SRUE trait. Bar, 10 mm

Favorable alleles carried by the superior parents for SRUE and corresponding phenotypic effect were summarized in Table 6. According to the phenotypic values and the number of favorable alleles that could be substituted or pyramided into an individual plant, the top 5 cross combinations predicted for SRUE and corresponding phenotypic increment effect (%) are listed in Table 7. For example, after crossing Yue40 × Manyedao, thirteen favorable alleles predicted could be pyramided into a single genotype, which led to a 0.16 mg.mg^− 1^ increase in SRUE value (Table 7). Certain accessions were found repeatedly in these proposed parental combinations (For example, Daniaodao), indicating that these accessions possess unique favorable alleles. Fig. 4 shows phenotypes of seeds of the superior parents and Fig. 5 shows the 10 days-old etiolated seedlings of the superior parents (Daniaodao, Manyedao, Suwujing, Yue 40 and Baimangnuo).

**Table 6:** Favorable alleles carried by the superior parents for SRUE and corresponding phenotypic effect

Code	Parental name	Favorable marker alleles and their phenotypic effect value (mg.mg^−1^)		
AL048	Daniaodao	RM128–165 (0.0890)	RM297–175 (0.4407)	RM5746–180 (0.0723)
		RM184–215 (0.4157)	RM3471–185 (0.0846)	
		RM232–160 (0.0751)	RM3589–100 (0.0593)	
		RM281–135 (0.0748)	RM525–135 (0.0691)	
AL051	Manyedao	RM128–165 (0.0890)	RM3138–110 (0.0517)	RM5818–150 (0.0836)
		RM184–205 (0.3795)	RM434–150 (0.1451)	RM7309–135 (0.1010)
		RM281–140 (0.1503)	RM525–135 (0.0691)	
		RM297–180 (0.4496)	RM5746–170 (0.1270)	
AL053	Baimangnuo	RM128–165 (0.0890)	RM3138–100 (0.0527)	RM5818–155 (0.0651)
		RM184–215 (0.4157)	RM434–150 (0.1451)	RM7309–135 (0.1010)
		RM281–140 (0.1503)	RM5746–175 (0.0929)	
AL113	Suwujing	RM232–160 (0.0751)	RM3471–135 (0.0573)	RM5818–155 (0.0651)
		RM281–145 (0.1019)	RM3589–100 (0.0593)	RM7309–160 (0.0580)
		RM3138–100 (0.0527)	RM434–155 (0.1284)	
AL311	Yue40	RM128–150 (0.0792)	RM3138–110 (0.0517)	RM434–150 (0.1451)
		RM232–120 (0.0973)	RM3471–110 (0.1332)	RM5818–145 (0.1305)
		RM281–135 (0.0748)	RM3589–220 (0.1143)	RM7309–115 (0.0802)

**Table 7:** Prediction of optimal parental combinations, favorable allele number and increment for SRUE after pyramiding

Code	Parental combinations	No. of positive favorable alleles predicted after crossing	SRUE increased after pyramiding (mg.mg^−1^)
AL311 × AL051	Yue40 × Manyedao	13	0.16
AL311 × AL048	Yue40 × Daniaodao	12	0.15
AL051 × AL113	Manyedao × Suwujing	13	0.14
AL113 × AL053	Suwujing × Baimangnuo	13	0.14
AL048 × AL113	Daniaodao × Suwujing	13	0.13

### Difference of seedling establishment rates between accessions with high and low SRUE in soil condition

**Fig. 6:**
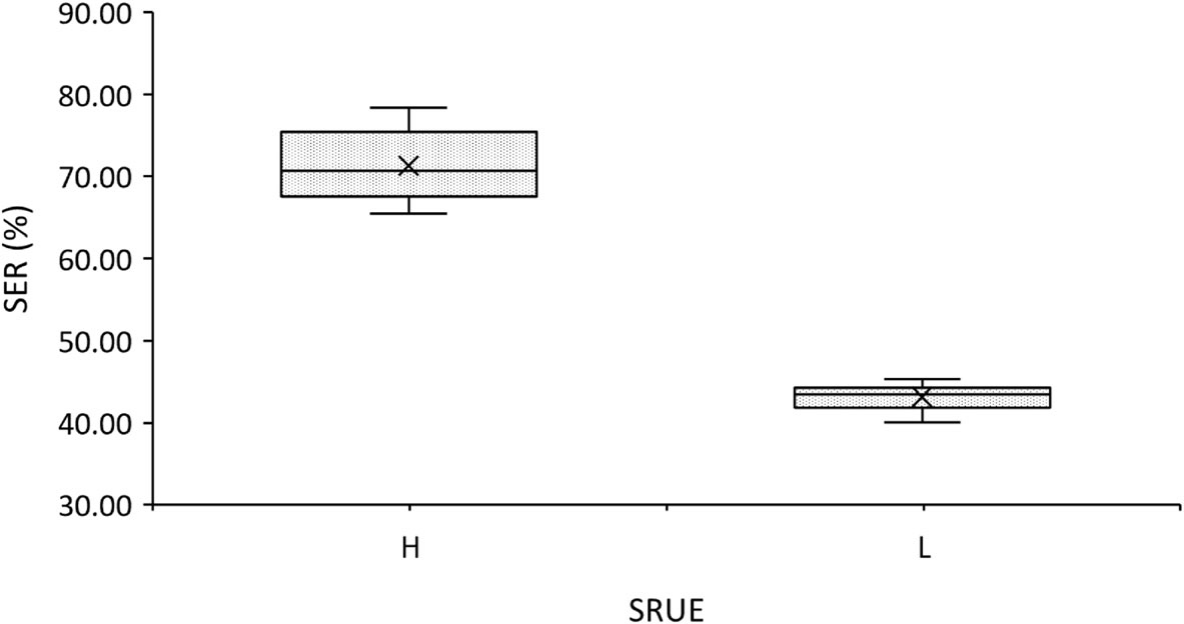
SER (%) bar graphic (with 95% CIs)

An experiment in soil condition was conducted to ascertain and confirm that the accessions with higher SRUE obtained in a growth chamber has a higher seedling establishment rate (SER) in soil cultivation condition. Under the soil trial, 42 selected accessions were divided into two groups, the first group comprised of accessions with high SRUE values (*n* = 22) and the second group comprised of accessions with low SRUE values (*n* = 20). The seeds were sown for a period of 15 days and kept under close observation. The number of established seedlings were recoded at the end of the trail period and SER(%) was calculated. The high SRUE group had numerically higher SER (%) than that of the low SRUE group. To determine if the effect of SRUE on SER was significat, an independent samples *t*-test was conducted. Table 8 show that there was a significant difference (*P* < 0.01) between the high SRUE varieties group (71.28 ± 4.22) and the low SRUE value varieties group (43.15 ± 1.54) in SER values; *t*_(27)_ = 29.23, *P* = 0.000. Therefore, the high SRUE varieties have statistically significantly higher SER values than the low SRUE varieties. The conclusion is that different SRUE values show significant differences in SER (%) and higher SRUE improved the SER. Fig. 6 represents the mean and the 95% confidence intervals for SER.

**Table 8:** Comparison of SER (%) between high and low SRUE (mg.mg^−1^) groups in the soil experiment

Variable	Mean (SD)		Mean difference (95% CI)	*t*-statistic (df)	*P*-value
SER^a^ (%)	High SRUE ^b^*n* = 22	Low SRUE *n* = 20	28.14 (26.16, 30.11)	29.23 (27)	0.000**
	71.28 (4.22)	43.15 (1.54)			

a SER rate: seedling establishment rate (%)

b SRUE: seed reserve utilization efficiency (mg.mg^−1^)

** *p* < 0.01

## Discussion

There were large variations in SRUE in natural population of rice used in this study. This is related to the wide geographic distribution of accessions used.The accessions were selected from 17° N in Vietnam to 54° N in northeast China, spanning 37° latitudes. And the large variations in SRUE are also related to the range of accession types, which included local varieties, modern bred varieties, high-stalk precocity varieties, and high-quality late maturing varieties. In addition, the two-year generalized heritability for SRUE is greater than 95%, indicating the variation of the trait was mainly controlled by genes and less affected by the environments. Therefore, molecular marker-assisted selection technologies can be used to improve SRUE trait for wet direct seeding.

In the soil trial, there was a significant different in SER (%) between the high and low SRUE groups at *P* = 0.01 (Table 8). The results indicate that accessions with high SRUE obtained from the growth chamber experiment had higher SER (%) under the soil conditions compared with the low SRUE. This suggests that SRUE is an important trait for seedling establishment rate. Although the soil trial is vital in confirming the accessions ability to emerge in the field, the growth chamber trial is a simpler and a more direct method for crop breeders to screen desirable germplasms for SRUE.

Population genetic structure is a substantive element in association studies that focus on traits that are important in local adaptation or diversifying selection with recent co-ancestry [29]. Using STRUCUTURE software and the neighbor- joining methods, the population used was divided into six subpopulations tied to the geographical origin. For example, POP1 accessions were from Jiangsu province, China and Vietnam, POP2 has accessions mainly from modern cultivars bred in north-central Jiangsu. This agreement between the genetic background and predefined clusters suggests that knowledge of the ancestral background can facilitate choices of parental lines in rice breeding programs [11, 13].

The accessions in the natural population have experienced a particular geographical isolation, and therefore there will be subpopulations with their own characteristics in the genetic composition, and genetic differentiation among the total populations. *F*_*st*_, fixed index refers to whether the actual frequency of genotype in the population deviates from the ratio of genetic equilibrium. Therefore, *F*_*st*_ can be used to compare the genetic differentiation between the two subpopulations, and then identify the genetic differences among varieties. In this study, the *F*_*st*_ values and the genetic distance between POP3 and POP4 were the largest among the other pairs of subpopulations. Agrama et al. (2007) [13] confirmed that markers with higher *F*_*st*_ values have greater resolving power and produce more consistent genetic distance estimates and the significant *F*_*st*_ among the subpopulations represents a real difference between them. Therefore, hybridization among subpopulations with different *F*_*st*_ values is possible to improve the trait value. Genome-wide analysis of the genetic diversity of 506 rice accessions using 266 SSR markers showed that 74% of the marker loci showed genetic diversity value larger than 0.7, with an average of 0.74. It was higher than 0.64 for Borba et al. (2009) [30], 0.73 for Dang et al. (2014, 2015) [20, 24] and 0.53 of Liu et al. (2015) [21]. However, it is less than 0.75 of Li et al. (2012) [16]. The average polymorphism information content was 0.71, this figure is higher than the 0.37 of Ordonez et al. (2010) [31], higher than the 0.48 of Liu et al. (2015) [21] and 0.70 of Dang et al. (2015) [20]; similar to Dang et al. (2014) [24] and Li et al. (2012) [16] and less than the 0.75 of Borba et al. (2009) [30]. More than 56% of the marker loci showed more than 10 alleles, with the average number of alleles per locus equal to 10.82, ranging from 2 (RM437_chromosome5, RM7163_chromosome11) to 38 (RM3428_chromosome11). The number of alleles per locus in our study was higher than that reported in Vanniarajan et al. (2012) 2.5 [17], Liu et al. (2015) 9.93 [21], Dang et al. (2014) 10.52 [24], and Dang et al. (2015) 10.40 [20], and less than those reported by Borba et al. (2009) 12.86 [30]. This variation may be due to the fact that the materials in the present study span from a wide geographical area stretching from north-central Vietnam to the northeastern part of China. In different climates and geographical conditions, the natural population experienced long-term natural selection and evolution, as well as different cultivation and management practices, have accumulated a high degree of genetic variation and a rich genetic background.

Linkage disequilibrium (LD) is the basis of association analysis. In comparison to other populations, the attenuation distances of POP2, POP3 and POP4 (27.75 cM, 17.57 cM and 19.23 cM, respectively) were consistent with the attenuation distances of 10 cM–30 cM reported by Vanniarajan et al. (2012) [17]. The attenuation of other subpopulations ranged from30cM to 60 cM. The extent of LD attenuation has been reported in rice [13, 17, 24, 32–35] but the results are quite different. For example, Olsen et al. (2006) [35] and Mather et al. (2007) [36] detect LD attenuation distances of less than 1 cM through DNA sequence. Jin et al. (2010) [37] detected LD attenuation distances of 25–50 cM using SSR markers. This difference is believed to be related to different genetic regions, different rice varieties and different markers [34, 36]. Therefore, the factors that affect the decay rate of LD are: population size, population source, number of loci and artificial selection. Based on the LD decay range in this population, genome wide LD mapping is possible. In this study, distances of LD decay of the 6 sub-populations were from 17.57 cM to 58.08 cM (Additional file 2: Figure. S2). This may suggest that 266 SSR markers are enough to detect significant loci associated with phenotypic variation of SURE in GWAS. However, to detect high-reliability and a greater number of significant loci in GWAS for SURE, it would be important to increase marker density and population size in the future experiments.

The association mapping helps to utilize the genetic variation in natural populations [38]. However, the population genetic structure and unequal relatedness among individuals could increase the false discoveries and lead to spurious associations. GLM consider only Q matrix generated during the study of population structure while MLM accounts for both population structure and the kinship (genetic relatedness among individuals) so generally GLM will detect higher number of significant marker-trait associations than MLM [39], Alternatively, MLM is more accurate in claiming associations than GLM, it had statistical advantage and detected more true associations than GLM [40]. In the current study, thirteen sites on chromosomes were found to be significantly associated with SRUE (PVE > 5%) and 23 favorable alleles (PEV > 0.1 mg.mg^− 1^) were detected in two years (Table 4 and Table 5).

RM 297 on chromosome 1 explained the maximum phenotypic variation, 34 accessions (6.72%) out of 506 carried excellent allele RM297–180 bp, with the largest phenotypic effect (0.44 mg.mg^1^ in 2016 and 0.45 mg.mg^− 1^ in 2017) and the typical carrier was Manyedao. Fifty-eight accessions (11.46%) carried excellent allele RM297–175 bp, with Daniaodao as a typical carrier. Followed by RM184 on chromosome 10 located at 41.6 cM, 30 (5.93%) and 51 (10.08%) accessions showed an excellent allelic variation of RM184–225 bp and RM184–215 bp, respectively and the typical carriers are Yandao6 and Daniaoda. Comparing with previous studies, Cheng et al. (2013) detected qSRUE1 interval (41166774–43,043,114 bp) with 10Mbp different from RM128 (30737705-30,737,861 bp). The interval of qSRUE4.1 (688353–2,030,305 bp) is 4Mbp different from RM 3471 (6310055-6,310,203 bp); the interval of qSRUE4.2 (2030135-8,067,386 bp) included RM3471 (6310055-6,310,203 bp). The interval of qSRUE6 (28,149,879 bp) is 2 Mb different from RM7309 (26297238-26,297,595 bp) on chromosome 6 [9]. RM297 (32099566-32,099,760 bp) on chromosome 1 has been identified by Cairns et al. (2009) to be related to the shoot length [41]. RM525 on chromosome 2 is located in the region (28292005-28,292,040 bp) in which a QTL for seedling dry weight has been detected by Han et al., (2007) [42]. RM232 on chromosome 3 is located in the region (15644275–15,646,800 bp) in which a QTLs for germination rate, seed weight, shoot length and root length has been detected in different studies [43–45]. RM434 on chromosome 9 is located in the region (15662573-15,662,838 bp) in which a QTLs for seedling dry weight has been detected in different studies [9, 43]. These results confirm the close relationship between seed and seedling traits with SRUE. In addition, SRUE could be enhanced by the crosses listed in Table 7, which shows cross combinations of accessions with complementary allelic variation at different loci to be selected as hybridization parents. The results of the current study provide basic marker information and accession information for breeding cultivars suitable to wet direct seeding by machine.

## Conclusions

There is abundant phenotypic variation for SRUE and molecular marker allele diversity among the 542 accessions used. Twenty-three favorable alleles for SRUE were detected across 2 years. Daniaodao, Manyedao, SuWujing, Yue 40 and Baimangnuo are the 5 typical carrier accessions possessing the favorable alleles. These accessions could be used to improve SRUE traits for mechanized live broadcasts.

## Methods

### Plant materials

The tested materials were 542 rice accessions^1^ [46]; 121 of which were from Vietnam (*Indica*), while the remaining accessions were from China (*Tej*). These accessions range from 17° N to 54° N and 102° E to135° E, crossing 37° latitude from the north to the south and 33° longitude from the east to west (Additional file 6: Table S2).

### Field planting

All the seeds of tested materials were sown in the seedling nursery of paddy fields in Jiangpu Experiment Station, Nanjing Agricultural University, in mid May 2016 and transplanted in mid-June. For each variety, four rows were transplanted. Each row had 8 hills with a spacing of 17 cm × 20 cm. Conventional field management practices were applied as recommended. In 2017, the dates of sowing and transplanting, and field management practices were identical to those in 2016.

### Phenotypic data collection (the growth chamber test)

Seeds of the natural population were harvested from the middle row of the plot at maturity stage and placed in a 50 °C oven for 72 h to break dormancy. The SRUE experiment was conducted in two replications for each season.

50 grains of healthy seeds of equal size, fullness and color were weighted to obtain the fresh weight (FW), then dried at 104 °C for 24 h to obtain the dry weight (DW). The water content (WC) was calculated using the following formula

$$ WC=\frac{FW- DW}{DW} $$

The initial seed dry weight (ISDW) was then calculated using the following formula

$$ ISDW=\frac{FW}{1+ WC} $$

SRUE was determined following the method described by Soltani et al. (2006) [2] and Cheng et al. (2013) [9] with minor modification. 50 seeds of each accession were lined up on a filter paper with 30 cm × 45 cm in size (Additional file 3: Figure S3a). The seeds were covered with two layers of moist filter paper and the papers rolled up and sealed with a rubber band (Additional file 3: Figure S3b). One end of the paper roll was covered with a self-sealing plastic bag and the other end of the paper roll was placed vertically in a plastic box (45.5 cm × 31.5 cm × 15 cm) with tap water of 10 cm depth (Additional file 3: Figure S3c). The plastic boxes were put in a growth chamber (GXZ and RXZ intelligent light incubator, Ningbo science and technology park, new Jiangnan instrument Co., Ltd., Ningbo, China) to germinate under complete dark condition and 30 °C for 10 days. During the period of germination, tap water was added to the plastic boxes to keep the paper roll moist. After 10 days, the etiolated seedlings (Additional file 3: Figure S3d) were separated into two parts, one including shoot and root, and the other including the seed remnant (Additional file 3: Figure S3e). Each part was dried at 105 °C for at least 24 h to obtain constant seedling dry weight (SDW) and the remnant seed dry weight (RSDW) (Additional file 3: Figure S3f). The following parameters were calculated based on the formula described by Cheng et al. (2013) [9].

The weight of mobilized seed reserve (WMSR)

$$ WMSR= ISDW- RSDW $$

Where ISDW is Initial seed dry weight.

Seed reserve utilization efficiency (SRUE)

$$ SRUE=\frac{SDW}{WMSR} $$

### Marker genotype identification

The plant leaves of the each accession in the natural population were collected 3 months after germination, and the total DNA was extracted using the method described by Murray and Thompson (1980) [47]. Marker genotype of each accession was identified using 266 pairs of SSR marker covering the 12 chromosomes in rice. The DNA sequence information of the 266 pairs of primers was obtained from the rice genome database (http://www.gramene.org) and was synthesized by Shanghai Jierui Biology Co., Shanghai, China.

Each 10 μL PCR reaction solution contained 1 μL template DNA (20 ng μL^− 1^), 0.7 μL forward primer (2 pmolμL^− 1^), 0.7 μL backward primer (2 pmolμL^− 1^), 1 μL 10 × Buffer (free MgCl_2_), 0.2 μL dNTP (2.5 m mol L^− 1^), 0.6 μL MgCl_2_ (25 m mol L^− 1^), 0.1 μLTaq (5 U μL^− 1^) and 6.4 μL ddH_2_O. The reaction procedure was carried out on a PTC-100 Peltier Thermal Cycler (MJ Research Inc., USA) with the program set to: (1) denaturation at 94 °C for 5 min; (2) 34 cycles of denaturation at 94 °C for 0.5 min, annealing at 55~61 °C (depending on primer used) for 1 min, and extension at 72 °C for 1 min; and (3) a final extension at 72 °C for 10 min. The PCR amplified product was run on 8.0% polyacrylamide gel (PAG). A DNA marker with a gradient of 100 bp was used as the control. The electrophoresis was done using 0.5X TBE buffer on 180 V constant voltage and then visualized using silver staining. Different sizes of DNA fragments amplified by the same pair of SSR primers were regarded as allelic variation fragments of the pair of primers and measured using software Quantity One.

### Population genetic structure and phylogenesis

Using STRUCTURE version 2.2 [48] the genetic clusters of the 542 accessions were identified. Five independent runs were performed for each K (K from 2 to 10). The length of the burn-in period was set to 50,000 iterations and defined a run of 100,000 Markov Chain Monte Carlo (MCMC) replicates after burn in. A mean log-likelihood value over five runs at each K was used. If the mean log-likelihood value was positively correlated with the model parameter K; the optimal K value was determined through an ad hoc statistic (∆K) based on the rate of change in [LnP(D)] between successive K values [26]. Non- admixed individuals in each genetic group were determined using a Q-matrix assignment greater than 0.9. Power Marker version 3.25 [49] was used to determine the number of alleles per locus, major allele frequency, genetic diversity per locus, and polymorphism information content (PIC) values per locus. The genetic distance was calculated based on 266 molecular markers using Nei’s distance [27] and phylogenetic reconstruction was performed using neighbor-joining method as implemented in Power Marker with the tree viewed using MEGA 4.0 [50]. Locus-by-locus analysis of molecular variance (AMOVA) [51] based on genetic groups delimited by the Bayesian clustering method in the program Arlequin 3.5 [52] was performed to statistically verify the structure using SSR and standard multi-locus frequency data. The genetic differentiation coefficient (*F*_st_) between subpopulation was calculated using the method proposed by Weir and Hill (2002) [53]. The calculation process was performed in Arlequin 3.5 software.

### Linkage disequilibrium

The linkage disequilibrium (LD) analysis was performed with TASSEL 2.1 software using 100,000 permutations to measure the level of linkage disequilibrium (LD) between loci [54], on all accessions and on the sub-populations generated by STRUCTURE. LD decay plot was drawn to observe the relationship between LD and genetic distance of syntenic (intra-chromosome).

### Phenotypic data analysis and heritability in a broad sense

Analysis of variance (ANOVA) was run to establish the genotypic and environmental variances among the traits measured using EXCEL 2013 software and the SAS package (SAS Institute Inc., CARY, NC, USA). Heritability in a broad sense (${H}_B^2$) was computed for the natural population using the following equation

$$ {H}_B^2={\sigma}_g^2/\left({\sigma}_g^2+{\sigma}_e^2/\mathcal{n}\right) $$

where ${\sigma}_g^2$ is genetic variance, ${\sigma}_e^2$ is error variance, and *퓃* is a number of replicates.

### Association mapping

The associations between the trait and the markers were analyzed by both general linear model (GLM) and mixed linear model (MLM) using TASSEL 3.0 software [54]. The Q matrix obtained from the analysis results of Structure 2.2 was used as covariant in the GLM analysis; while the matrices Q and K were used as covariates in the MLM analysis [24]. The K matrix (kinship matrix) was obtained from the results of the relatedness analysis using SPAGeDi software [55]. A false discovery rate (FDR) of 0.01 was used as a threshold for significant associations according to the correction method published by Benjamini and Hochberg (1995) [56]. Using the association locus identified, the “null allele” (non-amplified allele) was used to determine the phenotypic effects of the alleles [12]. The formula used for calculating phenotypic effect of a single allele was

$$ {a}_i=\sum {x}_{ij}/{n}_i-\sum {N}_k/{n}_K $$

where *a*_*i*_ was the phenotypic effect of the allele of *i*; *x*_*ij*_ denotes the phenotypic measurement values of *j* variety carrying the allele of *i*; *n*_*i*_ represents the number of materials carrying the allele of *i*; *N*_*k*_ denotes the phenotypic value of the variety of *k* carrying the null allele; and *n*_*K*_ represents the number of materials carrying the null allele. In the present study, marker loci with PVE > 5% were considered for further analysis. Varieties with higher phenotypic values together with the selected marker loci were analyzed to determine favorable alleles and their carrier accessions.

### Difference of seedling establishment rates in soil condition

Twenty-two varieties with high SRUE value and 20 varieties with low SRUE value were selected to confirm the results obtained from growth chamber through soil cultivation. Fifty healthy seed of each variety were used to germinate under room condition using the paper towel method, only sprouted seeds were used to conduct the soil cultivation (Additional file 4: Figure. S4).

The soil cultivation experiments were conducted in plastic cups (12 cm height × 9 cm diameter) with 2 mm (diameter) drainage holes at the bottom of the cups. The cups were filled with 11 cm of soil and tap water was added to saturate the soil. 30 sprouted seed of each variety were laid out on the surface and covered with 1 cm of soil. The cups were submerged under 2 cm of water in plastic boxes (45.5 cm × 31.5 cm × 15 cm) and left to grow for 15 days under the soil conditions. A plastic cover was used to protect the germinated seeds from the birds and rain splash damage. The experiment was conducted in three replications.

Out of 30 sprouted seeds, the number of established seedlings was counted and the percentage of seedling establishment was calculated using the following formula described by Islam et al., 2014 [57]:

$$ Seedling\ establishment\ rate\left(\%\right)=\frac{Number\ of\ establishment\ plants}{Number\ of\ total\ seedling}\times 100 $$

## Supplementary information

**Additional file 1. Figure S1.** Gel picture display SSR profiles amplified by primer RM3428 using total DNA as template. 1: Yingtoudao; 2: Changdaotou; 3: Yangmiaozhong; 4: Maoguangdao; 5: Dazhongdao; 6: Sanxiadao; 7: Xiaoqingmang; 8: Hongganlizhihong; 9: Wuxidao; 10: Wanzhognqiu; 11: Fengjingdao; 12: Liuzhong; 13: Cuganlizhihong; 14: Chiguwandao; 15: Jiaobaiyeqing; 16: Chiguhong; 17: Fanluoqing; 18: Zaoyedao; 19: Baidiegu; 20: Wangjiadao; 21: Jiangyinzhong; 22: Eyingbaijingdao; 23: Tiekewanguangtou; 24: Tiekedao; 25: Dadaosuitou; 26: Aibaidao; 27: Xiepihuang; 28: Xiaobaidao; 29: Baishidao; 30: Manbaidao; 31: Guangtouluhuabai; 32: Hongmangjing; 33: Wumangyedao; 34: Luhuabai; 35: Haidongqing; 36: Shenlenuo; 37: Xiangqing; 38: Jinghui418; 39: Malaihong; 40: Jingnuo330; 41: Zaijinjing; 42: Fuyu3; 43: Dongnongjing424; 44: Dongnongjingnuo418; 45: R254; 46: Jiangyinnuo; 47: Jinggunuo; 48: Shanhonggu

**Additional file 2. Figure S2.** Relationship between D ‘values and genetic distances of syntenic (intra-chromosome) marker pairs in six sub-populations

**Additional file 3. Figure S3.** Part of the experiment operation process of SRUE measurement. a. Rice grains were lined on the filter papers. b. Rolled the papers and sail it with rubber band. c. Cover the top of the paper roll with self-sealing plastic bag and then vertically place them into a plastic box containing a layer tap water (10 cm depth). d. Etiolated seedlings after 10 days’ culture under complete dark at 30 °C. e. Separated fresh etiolated seedling (shoot and root) and the grain remnant on aluminum foil. f. Dried etiolated seedling (shoot and root) and grain remnant on aluminum foil.

**Additional file 4. Figure S4.** Partial soil experiment operation process for SRUE measurement. A. Rice grains priming. B. Lying the germinated seed in the soil. C. e seed under the soil conditions in the boxes. D. Comparison of SER performance between high and low SRUE varieties under the soil conditions. E. Seedlings of the superior parents after 15 days’ culture under the soil conditions.

**Additional file 5. Table S1.** Summary statistics for the 266 SSR markers used in this study.

**Additional file 6. Table S2.** The code, name and origin of 542 rice accessions and the Q value of each accession belonging to the 6 subpopulations in this study.

**Additional file 7. Table S3.** Analysis of molecular variance (AMOVA) for six subpopulations of rice accessions.

**Additional file 8. Table S4.** Comparisons of marker loci detected in this study with loci reported previously.

## Data Availability

The rice accessions names and geographical origins are available in Additional file 5: Table S1.

## Abbreviations

cM: Centi-Morgan
dNTP: Deoxyribonucleoside trisphosphate
DW: Dry weight
EDTA: Disodium ethylenediaminetetra-acetate
FDR: False discovery rate
FW: Fresh weight
GLM: General linear model
*H*^*2*^_*B*_: Heritability in the broad sense
ISDW: Initial seed dry weight
LD: Linkage disequilibrium
MCMC: Markov Chain Monte Carlo
MLM: Mixed linear model
PCR: Polymerase Chain Reaction
PIC: Polymorphic information content
QTL: Quantitative trait loci
RSDW: Remaining seed dry weight
SDS: Sodium dodecyl sulfate
SDW: Seedling dry weight
SRUE: Seed reserve utilization efficiency
SSR: Simple sequence repeat
Taq: Thermus aquaticus DNA polymerase
TBE: Tris/borate electrophoresis buffer
TEMED: Tetramethylenediamine
Tris: Tris (hydroaymethyl) aminonethane
WC: Water content
WMSR: Weight of the mobilized seed reserve

## Footnotes

1 All the rice seeds used in this research were collected during long-term rice science studies and properly kept in our State Key Laboratory of Crop Genetics and Germplasm Enhancement, Nanjing Agricultural University. Accession numbers 1–542 were selected from our previous studies on rice grain sizes and weight (Rf. 10.3389/fpls.2016.00787).
