# Supplementary figures and images for: Mining of favorable alleles for seed reserve utilization efficiency in Oryza sativa by means of association mapping
Source: BMC Genet. 2020 Jan 16;21:4. doi: 10.1186/s12863-020-0811-3 (PMC6966888; doi:10.1186/s12863-020-0811-3)

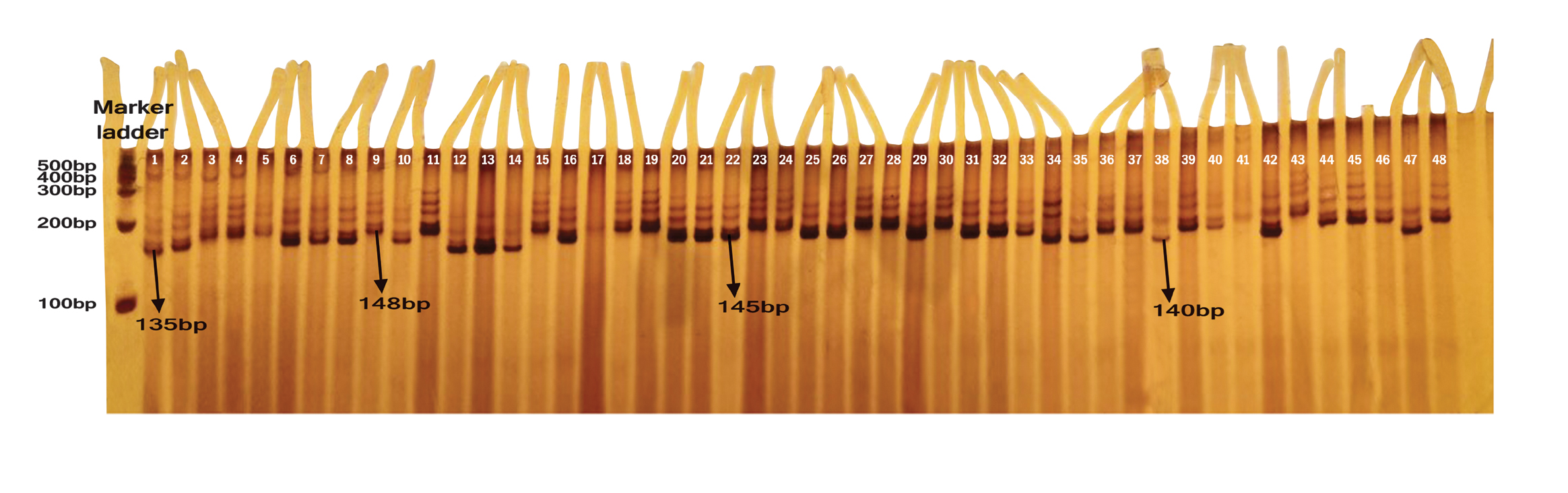

Supplement: Supplementary file 1 — Additional file 1. Figure S1. Gel picture display SSR profiles amplified by primer RM3428 using total DNA as template. 1: Yingtoudao; 2: Changdaotou; 3: Yangmiaozhong; 4: Maoguangdao; 5: Dazhongdao; 6: Sanxiadao; 7: Xiaoqingmang; 8: Hongganlizhihong; 9: Wuxidao; 10: Wanzhognqiu; 11: Fengjingdao; 12: Liuzhong; 13: Cuganlizhihong; 14: Chiguwandao; 15: Jiaobaiyeqing; 16: Chiguhong; 17: Fanluoqing; 18: Zaoyedao; 19: Baidiegu; 20: Wangjiadao; 21: Jiangyinzhong; 22: Eyingbaijingdao; 23: Tiekewanguangtou; 24: Tiekedao; 25: Dadaosuitou; 26: Aibaidao; 27: Xiepihuang; 28: Xiaobaidao; 29: Baishidao; 30: Manbaidao; 31: Guangtouluhuabai; 32: Hongmangjing; 33: Wumangyedao; 34: Luhuabai; 35: Haidongqing; 36: Shenlenuo; 37: Xiangqing; 38: Jinghui418; 39: Malaihong; 40: Jingnuo330; 41: Zaijinjing; 42: Fuyu3; 43: Dongnongjing424; 44: Dongnongjingnuo418; 45: R254; 46: Jiangyinnuo; 47: Jinggunuo; 48: Shanhonggu [file 12863_2020_811_MOESM1_ESM.jpg]

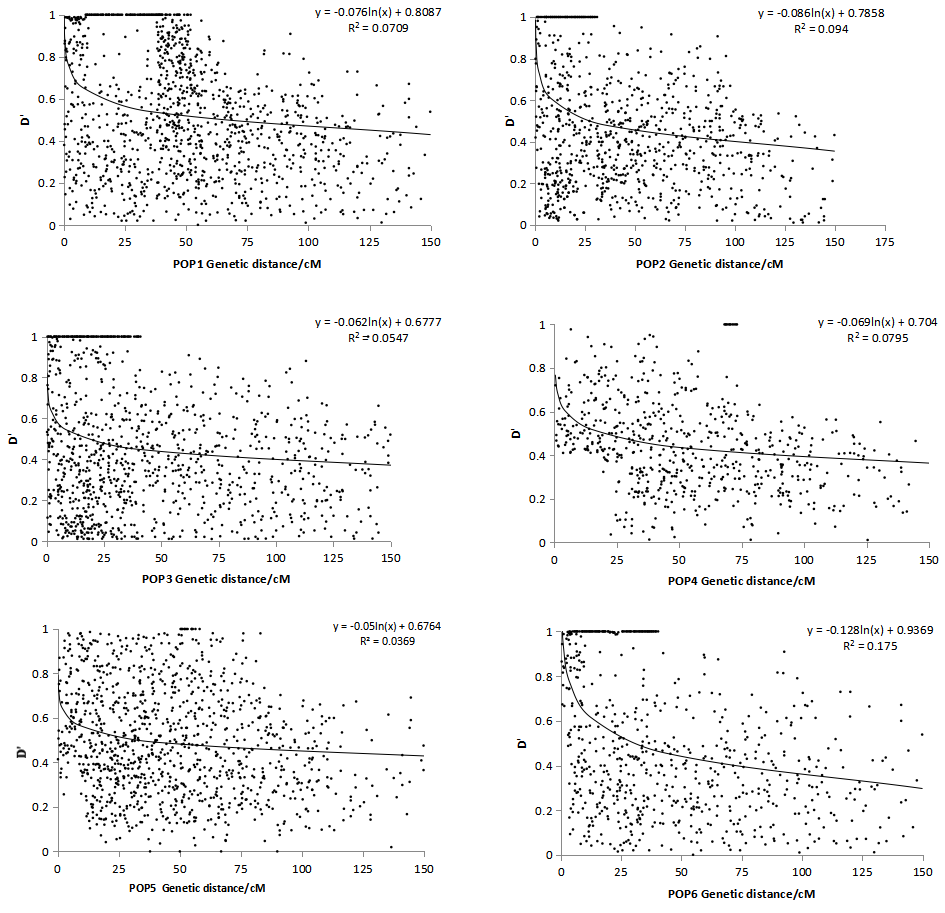

Supplement: Supplementary file 2 — Additional file 2. Figure S2. Relationship between D ‘values and genetic distances of syntenic (intra-chromosome) marker pairs in six sub-populations [file 12863_2020_811_MOESM2_ESM.tif]

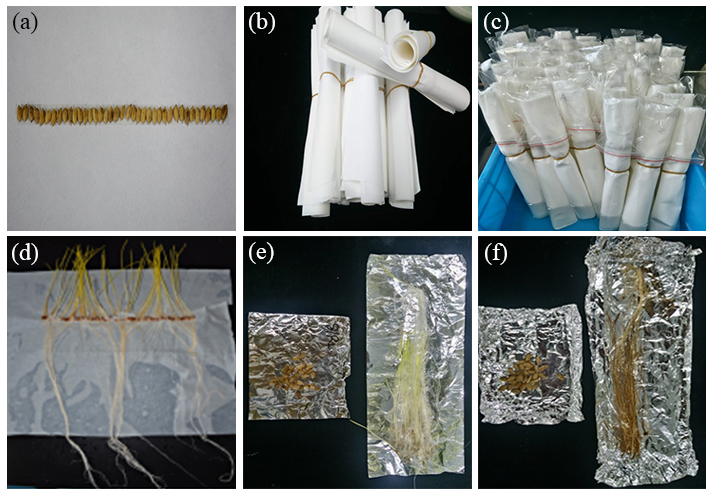

Supplement: Supplementary file 3 — Additional file 3. Figure S3. Part of the experiment operation process of SRUE measurement. a. Rice grains were lined on the filter papers. b. Rolled the papers and sail it with rubber band. c. Cover the top of the paper roll with self-sealing plastic bag and then vertically place them into a plastic box containing a layer tap water (10 cm depth). d. Etiolated seedlings after 10 days’ culture under complete dark at 30 °C. e. Separated fresh etiolated seedling (shoot and root) and the grain remnant on aluminum foil. f. Dried etiolated seedling (shoot and root) and grain remnant on aluminum foil. [file 12863_2020_811_MOESM3_ESM.jpg]

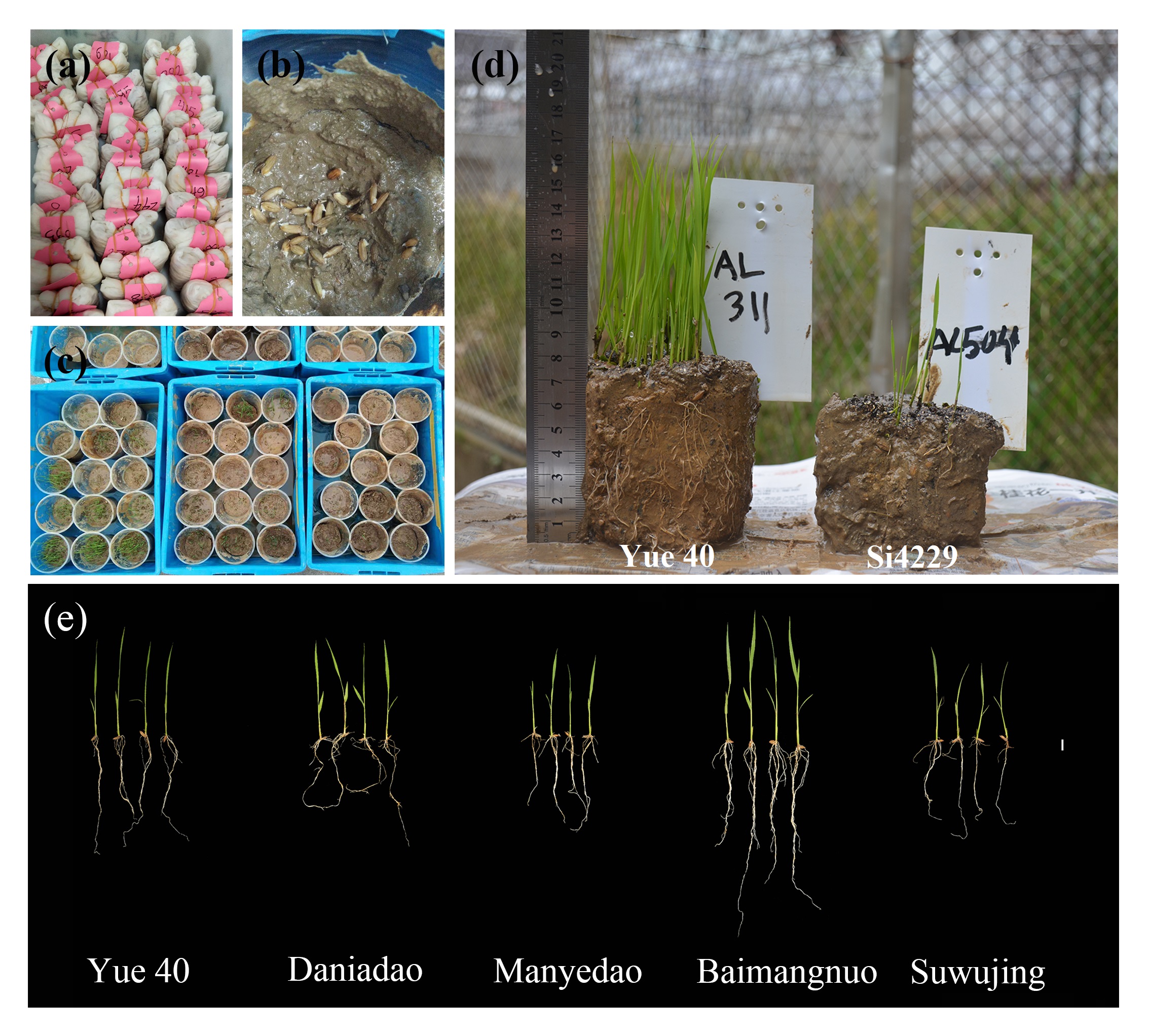

Supplement: Supplementary file 4 — Additional file 4. Figure S4. Partial soil experiment operation process for SRUE measurement. A. Rice grains priming. B. Lying the germinated seed in the soil. C. e seed under the soil conditions in the boxes. D. Comparison of SER performance between high and low SRUE varieties under the soil conditions. E. Seedlings of the superior parents after 15 days’ culture under the soil conditions. [file 12863_2020_811_MOESM4_ESM.jpg]
